# Differentiation of wild-type and *PAP1*-overexpressing tobacco by volatile organic compound profiling using TP-SESI mass spectrometry

**DOI:** 10.1007/s00216-026-06630-y

**Published:** 2026-06-20

**Authors:** Sarah M. Ashbacher, De-Yu Xie, David C. Muddiman

**Affiliations:** 1 Biological Imaging Laboratory for Disease and Exposure Research (BILDER), Department of Chemistry, North Carolina State University, Raleigh, NC 27695 USA; 2 Department of Plant and Microbial Biology, North Carolina State University, Raleigh, NC 27695 USA

**Keywords:** TP-SESI, VOCs, *PAP1*, Plant metabolomics, Mass spectrometry

## Abstract

Real-time profiling of plant volatiles provides insight into metabolic regulation, yet rapid and high-throughput characterization remains analytically challenging. Here, we apply temperature programming secondary electrospray ionization (TP-SESI) mass spectrometry to compare volatile organic compound (VOC) profiles from wild-type and *PAP1*-overexpressing tobacco (*Nicotiana tabacum*). This study extends the application of TP-SESI, which was originally developed for plant volatile analysis, to evaluate genotype-specific metabolic differences. TP-SESI enabled sensitive detection of VOCs and semi-volatile compounds spanning three major plant volatile classes: terpenoids, phenylpropanoids/benzenoids, and fatty acid derivatives, across both genotypes. While the two genotypes shared many detected features, *PAP1*-overexpressing plants were associated with upregulation of these indicated VOCs, suggesting broad metabolic reprogramming associated with *PAP1* activation. Statistical analyses revealed clear separation between genotypes, demonstrating TP-SESI captures robust, reproducible metabolic differences in intact plant tissue. These results suggest that TP-SESI is capable of detecting genotype-associated variations in plant volatile emissions and may be useful for examining transcription factor–related changes in plant metabolism.

**Graphical abstract:**

**Figure:**
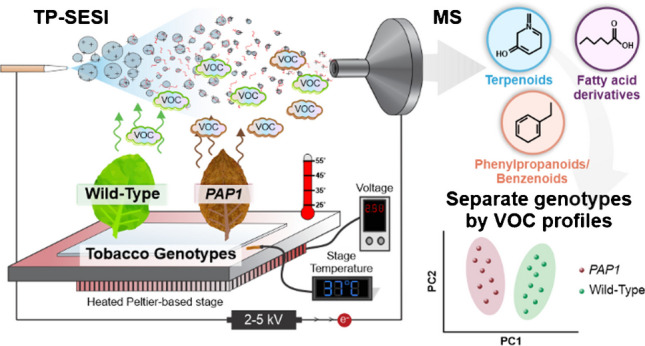

**Supplementary Information:**

The online version contains supplementary material available at 10.1007/s00216-026-06630-y.

## Introduction

Volatile organic compounds (VOCs) emitted by plants provide insight into their underlying metabolic activity and physiological state. These small molecules are produced as products of both primary metabolism, such as sugars, amino acids, and lipids, and secondary metabolism, including phenylpropanoids, terpenoids, and nitrogen-containing compounds [1–3]. Plant VOC profiles are highly dynamic and are influenced by genetic background, developmental stage, and environmental conditions, including temperature, light, and biotic or abiotic stress [4, 5]. Because VOCs often respond rapidly to changes in plant physiology and environmental conditions, they serve as sensitive indicators of metabolic changes, enabling early detection of developmental transitions, stress responses, and genotype-specific metabolic profiles [6].

As a result, analysis of plant VOCs has become an increasingly valuable tool for probing metabolism, assessing phenotypic variation, and studying ecological interactions, such as plant communication and defense signaling [7]. However, comprehensive characterization of plant VOCs remains analytically challenging due to their chemical diversity. Additional challenges arise from the wide dynamic range of concentrations, complex matrices, and the presence of structurally similar isomers, all of which can complicate accurate detection and identification [4, 8]. Collectively, these factors necessitate analytical approaches that can capture a broad range of compounds with high sensitivity, chemical specificity, and temporal resolution, while ideally preserving the biological context of the plant tissue.

Mass spectrometry–based techniques have played a central role in advancing VOC analysis. Gas chromatography mass spectrometry (GC-MS) remains the gold standard for VOC identification but often requires extensive sample preparation, derivatization, and long analysis times, limiting throughput and real-time applicability [9, 10]. Ambient ionization mass spectrometry techniques provide a compelling alternative for plant metabolomics by enabling rapid, direct analysis with minimal sample preparation. For example, neutral desorption-extractive electrospray ionization mass spectrometry (ND-EESI-MS) enables direct in situ profiling of plant stress–related metabolites without sample preparation, with broad sensitivity toward both polar and moderately nonpolar compounds [11]. Secondary electrospray ionization (SESI) has also emerged as a powerful approach for detecting VOCs and semi-volatile organic compounds in complex biological systems [12, 13]. In SESI, gas-phase analytes are ionized through charge transfer reactions with electrospray-generated reagent ions, enabling soft ionization and high sensitivity without chromatographic separation [14].

SESI is highly sensitive to volatile compounds, but its detection efficiency decreases for metabolites that are less volatile or remain bound to the plant surface. These low-volatility or surface-associated metabolites are not readily released into the gas phase under ambient conditions, which can limit their detection in real-time analyses [15, 16]. Controlled thermal release offers an effective strategy to enhance the emission of such species, thereby expanding the range of detectable metabolites. Temperature programming secondary electrospray ionization (TP-SESI) further expands the chemical profiles accessible to SESI by promoting controlled thermal desorption of low- and semi-volatile metabolites [17]. Localized heating of the sample surface increases the vapor pressure of low-volatile compounds, facilitating their release into the gas phase without the need for extraction or chemical derivatization. This controlled thermal release enables access to metabolites that are otherwise difficult to detect by conventional SESI, including oxygen-rich species, conjugated secondary metabolites, and low-abundance intermediates [18]. Importantly, the added temperature factor can be tuned to systematically probe the relationship between thermal energy and metabolite emission, providing additional insight into compound volatility and desorption behavior. When coupled with high-resolution mass spectrometry, TP-SESI allows for sensitive, real-time detection of VOCs and semi-VOCs with high mass measurement accuracy, enabling confident annotations and comparative analysis across biological conditions. These features make TP-SESI particularly well suited for non-destructive, in situ characterization of plant metabolic phenotypes, where subtle genetic perturbations can lead to coordinated changes across multiple metabolic pathways.

The ability of TP-SESI to access a broad range of volatile and semi-volatile metabolites provides new opportunities for linking VOC profiles to underlying genetic regulation. Transcriptional regulators that reprogram secondary metabolism provide an ideal framework for evaluating the sensitivity and specificity of TP-SESI-based VOC profiling. One regulator is the MYB transcription factor *PAP1 *(Production of Anthocyanin Pigment 1), a member of the R2R3-MYB family, that functions as a central activator of phenylpropanoid and flavonoid biosynthetic pathways [19]. *PAP1 *has been shown to directly upregulate numerous structural genes in these pathways, resulting in enhanced accumulation of anthocyanins and other flavonoids across multiple plant species [20, 21]. Overexpression of *PAP1* in tobacco and other hosts leads to characteristic red or purple pigmentation and elevated levels of these phenylpropanoid metabolites, demonstrating its capacity to drive broad metabolic reprogramming beyond pigmentation alone. Additionally, *PAP1 *overexpression is influenced by environmental and signaling cues, such as sugar levels and light, reflecting its integration into wider regulatory networks that coordinate carbon allocation and stress responses [22, 23]. Because *PAP1* overexpression alters a wide array of downstream metabolites that span both volatile and semi-volatile classes, it acts as a biologically relevant model system to assess whether TP-SESI can detect genotype-specific differences in these VOC metabolic signatures.

In this study, we apply TP-SESI mass spectrometry to compare VOC and semi-VOC profiles of *PAP1*-overexpressing tobacco leaves and wild-type controls. Using a combination of univariate and multivariate statistical analyses, we evaluate the ability of TP-SESI to differentiate plant genotypes based on emitted chemical profiles. We further examine the putative chemical classes and metabolic pathways associated with differentially abundant annotations, with emphasis on pathway-level interpretation. Each TP-SESI measurement consists of continuous real-time acquisition during a controlled temperature program (~ 3 min per leaflet), with only minimal sample handling and no extraction, derivatization, or chromatographic separation, enabling a streamlined workflow in which throughput is governed by direct analysis time. This work demonstrates the capability of TP-SESI to resolve genotype-dependent metabolic differences in intact plant tissue and highlights its utility as a rapid, minimally invasive tool for plant metabolomics and phenotyping studies.

## Experimental

### Materials

LC-MS-grade acetonitrile (ACN), water (H₂O), and formic acid (all obtained from Fisher Scientific, Nazareth, PA, USA) were used to prepare the electrospray solvent. LC-MS-grade methanol was used to clean the scissors and tweezers between sampling, and plain 1-mm microscope glass slides from Fisher Scientific served as the mounting surface. Leaves were affixed to the slides using double-sided tape (AMZTP01, Amazon, Seattle, WA, USA).

Wild-type tobacco and *PAP1* overexpressed plants were 3 weeks old at the time of analysis. Seeds were germinated and grown in pots in a greenhouse under natural light at 25–30 °C. For each measurement, an intact leaflet was excised using methanol-sanitized scissors and mounted with tweezers on a glass slide for analysis.

### TP-SESI MS analysis

All TP-SESI experiments were conducted on the Next Generation IR-MALDESI source, coupled to an Orbitrap Exploris 240 mass spectrometer (Thermo Fisher Scientific, Bremen, Germany), as described in previous reports [24]. Mass spectra were acquired in positive ionization mode over an *m*/*z* range of 100–400. High mass measurement accuracy (± 2.5 ppm) was ensured using the EASY-IC internal calibration source (fluoranthene, [M●+] m/z 202.0777) in centroid mode. The instrument was operated at a resolving power of 240,000 FWHM (*m*/*z *200) with a fixed injection time of 15 ms [25], without automatic gain control (AGC). Daily mass calibration was performed prior to sample acquisition.

The sample stage was controlled with RastirZ software, allowing precise positioning of the leaflets during analysis. The electrospray emitter was aligned coaxially with the sample inlet, positioned 5 mm orthogonal to the stage to maximize ionization efficiency. The electrospray solution consisted of ACN:H₂O (50:50, v/v) with 0.2% formic acid, delivered at 1–2 µL/min and stabilized with a high voltage of 3–4 kV. Leaf samples were analyzed directly in real time, without prior extraction or derivatization.

For each treatment group (wild-type and *PAP1*-overexpressing tobacco plants), one individual plant was analyzed. Three independent leaflets were sampled from each plant and analyzed by TP-SESI. Leaflet collection and analysis were conducted in a randomly alternating order between genotypes to minimize systematic bias during acquisition. Each leaflet was analyzed using continuous TP-SESI acquisition, with the number of scans per leaflet ranging from approximately 50 to 250 depending on the experimental setup (e.g., temperature ramp or fixed-temperature acquisition). These scans were used for signal averaging and generation of VOC profiles. Thus, statistical analyses are based on leaflet-level measurements (*n* = 3 per genotype), with scans representing repeated instrumental measurements within each leaflet.

### Data processing

All raw data were collected as Thermo Fisher. RAW files and imported directly into MSiReader v3.12 (MSI Software Solutions, LLC, Raleigh, NC, USA) [26] for visualization and quantification. PCA and heatmaps were generated to explore patterns of metabolite abundance between wild-type and *PAP1* samples. Raw spectra for individual analytes of interest were examined using Freestyle v1.8 (XCalibur, Thermo Fisher Scientific Inc.). For putative compound annotation, METASPACE [27] was employed with the ChEBI database, using a false discovery rate (FDR) of 10% [28]. This threshold was selected to maximize the number of retained annotations while maintaining a reasonable level of confidence in putative VOC assignments. They were further cross-validated against two additional databases: vocBinBase [29] and KNApSAcK [30], and only compounds detected in at least two of the three databases were retained for subsequent analysis. Further statistical analyses, including boxplots and *t*-tests, were conducted in R v4.3.3 to quantify differences in metabolite levels between genotypes.

## Results and discussion

**Fig. 1:**
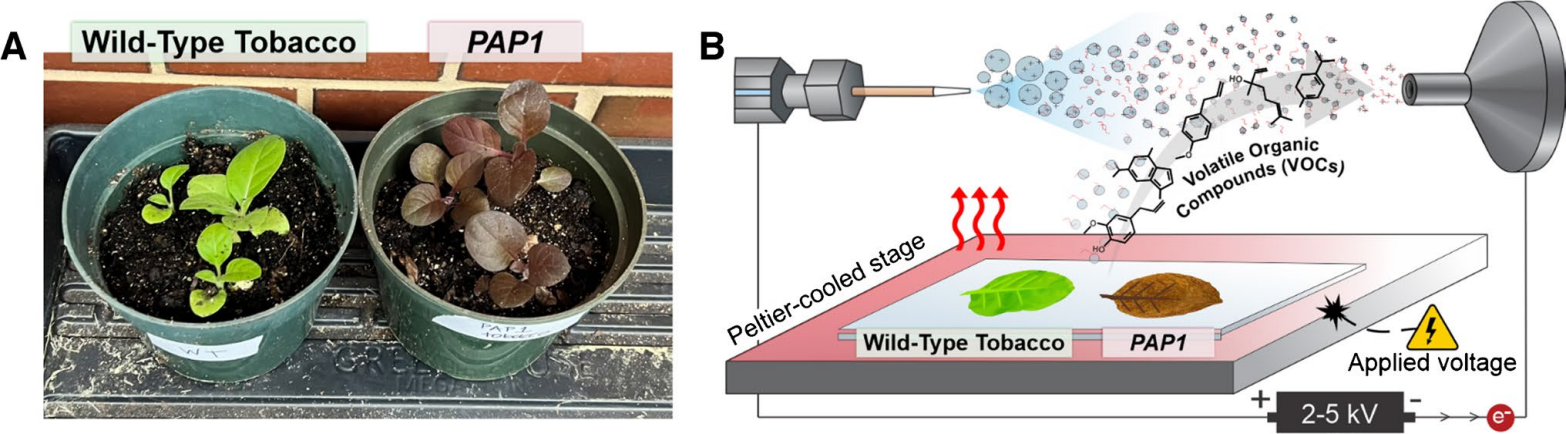
**A** Wild-type (left) and *PAP1* tobacco (right) plants showing the difference in color. The wild-type plant exhibits typical green pigmentation while the *PAP1* plant displays red/purple pigmentation due to anthocyanin accumulation. **B **TP-SESI workflow used for VOC analysis. Both plant types were analyzed separately on a temperature-controlled stage where temperature was regulated using a Peltier-based system (by an applied voltage), inducing release of volatile and semi-volatile metabolites. Emitted VOCs intersect the electrospray plume where gas-phase ionization occurs prior to entering the mass spectrometer for detection

The wild-type and *PAP1*-overexpressing tobacco plants displayed visually distinct phenotypes (Fig. 1A). The wild-type plant exhibits green pigmentation, typical of healthy tobacco, whereas the *PAP1*-overexpressing plant shows a red or purple pigmentation, consistent with anthocyanin production. This color difference reflects the transcriptional activation of the phenylpropanoid pathway by *PAP1*. The pronounced pigmentation in the *PAP1* plant provides a clear visual indicator of successful overexpression in order to successfully examine differences in VOC profiles.

To investigate how these genotype-dependent metabolic differences translate into VOC emissions, we applied TP-SESI to monitor volatile and semi-volatile compounds in real time under controlled temperature conditions. Figure 1B presents a schematic of the TP-SESI workflow used to analyze VOC emissions from wild-type and *PAP1*-overexpressing plants. This approach builds on a previous proof-of-concept study demonstrating the ability of TP-SESI to capture semi-volatile and thermally labile metabolites at different temperature points [17]. The experimental setup is designed to allow precise control of sample stage temperature while ensuring efficient analysis of emitted VOCs. Temperature adjustments were applied gradually using a Peltier system, enabling continuous monitoring of VOC profiles as a function of temperature. As the stage temperature increased, VOC emission was tracked in real time by recording ion abundance at each scan. TP-SESI leverages electrospray ionization in the gas phase where volatile molecules released from the sample collide with charged droplets, facilitating efficient ionization for mass spectrometric detection. This setup enables simultaneous monitoring of temperature-dependent VOC changes and provides the foundation for comparing metabolic differences between the two genotypes. For comparative analysis, VOC profiles from wild-type and *PAP1*-overexpressing plants were evaluated at a stage temperature of 55 °C, selected to enhance emission of volatile and semi-volatile metabolites. 55 °C was selected as the comparative condition because it yielded maximal and stable VOC signal across both genotypes within the explored temperature range. No evidence of temperature-induced artifacts or emergence of new dominant ion populations was observed across the investigated range, supporting thermal desorption rather than thermal degradation.

**Fig. 2:**
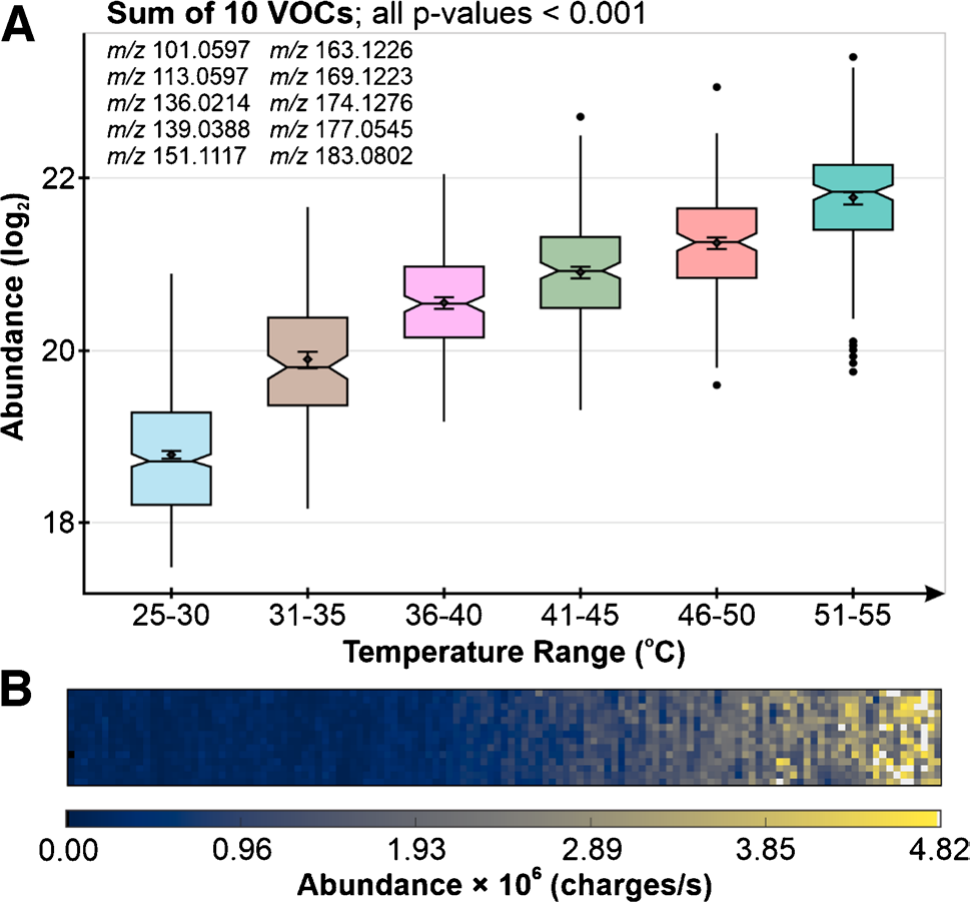
**A** Notched boxplots showing the summed ion abundance of 10 selected VOCs in *PAP1* across a controlled temperature range (25–55 °C, 5 °C intervals). Statistically significant differences were observed between all temperature ranges (*p* < 0.001), indicating strong temperature dependence of VOC emission. **B** Heatmap of the summed abundance of the same 10 VOCs as a function of stage temperature. Signal increases progressively with temperature supporting the boxplot results

Prior to comparing VOC emissions between *PAP1*-overexpressing and wild-type plants, we first verified that temperature ramping produced statistically distinguishable changes in VOC abundance within the *PAP1* samples. The effect of temperature on VOC emission was evaluated by monitoring 10 representative VOCs across a controlled stage temperature range of 25–55 °C. These analytes span the three major volatile classes, in addition to an “other” category for compounds that do not fall within those classifications (i.e., amino acid derivatives, heteroaromatics, etc.). Figure 2A presents boxplots of the summed abundances of these compounds at 5 °C intervals, revealing statistically significant differences between each temperature range (*p*-value < 0.001). VOC data were acquired continuously during the temperature ramp, with no equilibration between temperature changes. For analysis, the continuous dataset was post-binned into 5 °C temperature ranges. Each temperature value corresponds to approximately 50 consecutive scans (~28 s of acquisition) which equate to about 250 scans per temperature interval (~2.3 min). This progressive increase in abundance demonstrates that stage temperature is a dominant factor of VOC release and highlights the effectiveness of controlled thermal input for mobilizing both volatile and semi-volatile metabolites from leaf tissue. The steady increase in VOC levels with rising temperature indicates that higher heat gradually releases metabolites from the tissue by promoting thermal desorption and enhancing their diffusion. Figure 2B provides a complementary heatmap of the summed abundances of the same 10 VOCs, illustrating a clear and systematic increase in signal intensity as temperature rises. The concordance between the boxplot and heatmap reinforces the robustness of the observed temperature dependent trends. These selected ions reflect the dominant features observed in the full TP-SESI mass spectra over the same scan range and therefore serve as representative markers of the overall VOC response to temperature. The same monotonic increase in abundance were observed across the broader annotated VOC dataset, indicating that the selected ions accurately reflect the overall temperature-dependent spectral behavior of the volatile metabolome. By confirming that gradual heating produces predictable changes in VOC emission, these results establish a foundation for subsequent comparisons between wild-type and *PAP1*-overexpressing plants.

**Fig. 3:**
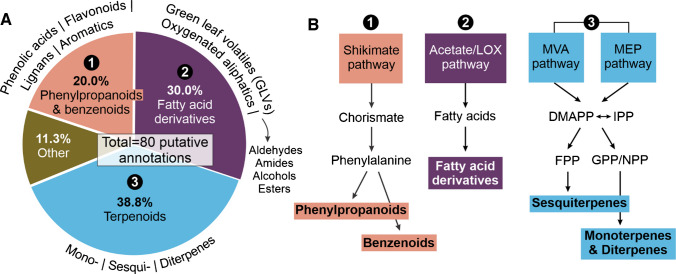
**A** Pie chart summarizing the distribution of 80 annotated VOCs detected by TP-SESI verified by multiple databases. Terpenoids comprise 38.8% of annotations, followed by fatty acid derivatives (30%) and phenylpropanoids/benzenoids (20%). The outer ring indicates subclasses associated with each major VOC category. **B** Simplified overview of the biosynthetic pathways of the three major VOC classes detected. 1, Phenylpropanoids/benzenoids (orange) originate from the shikimate pathway; 2, fatty acid derivatives (purple) arise primarily from the acetate and lipoxygenase (LOX) pathways; and 3, terpenoids (blue) derive from the mevalonate (MVA) and methylerythritol phosphate (MEP) pathways

To further interpret the metabolic composition accessed by TP-SESI, all detected VOCs were grouped according to their biosynthetic origin. Putative identifications for these analytes were cross-validated using METASPACE, the vocBinBase database, and the KNApSAcK metabolite database to strengthen confidence in structural annotation. All compounds are reported as putative annotations based on mass spectrometric and database evidence and should be interpreted at the metabolite-class level. This yielded 80 putative annotations in *PAP1*-overexpressing plants, which were curated for reliability (see Supplementary Materials). Annotations were only included in analysis if they were present in at least 2 out of 3 databases. These 80 VOCs were then classified into three primary categories, fatty acid derivatives, phenylpropanoids/benzenoids, and terpenoids, based on their metabolic precursors and core biosynthetic pathways [3, 7, 31]. As shown in Fig. 3A, terpenoids represent the largest fraction of detected VOCs, accounting for 38.8% of annotations. This group includes mono-, sesqui-, and diterpene-related species derived from the mevalonate (MVA) and methylerythritol phosphate (MEP) pathways. Terpenoids represent one of the most structurally diverse plant VOCs and are often key contributors to plant-environment interactions, including defense, signaling, and communication [32]. Fatty acid derivatives comprise 30% of the detected VOCs. This category encompasses a broad range of oxygenated aliphatics, including aldehydes, alcohols, esters, and related species commonly associated with membrane lipid metabolism and lipoxygenase-mediated oxidation via the acetate/LOX pathway [33, 34]. These metabolites are often released in response to wounding, herbivory, or oxidative stress, functioning as defense signals and modulators of plant-environment interactions, well documented as “green leaf volatiles” [35, 36].

Phenylpropanoids and benzenoids account for 20% of detected VOCs. These aromatic metabolites originate from the shikimate pathway [37, 38], and include phenolic acids, methoxylated aromatics, flavonoids, and related conjugates that play central roles in plant defense, pigmentation, and stress signaling. Their presence aligns with the use of *PAP1*-overexpressing plants in this study, as *PAP1 *is known to transcriptionally activate the phenylpropanoid pathway and broadly reprogram secondary metabolism [39]. The remaining 11.3% of annotations were categorized as “other,” representing metabolites that do not fall within the three dominant volatile classes. This group includes tobacco alkaloid–related compounds, like nicotine, characteristic of *Nicotiana *specialized secondary metabolism [40, 41]. Additional analytes comprised products associated with thermal or sugar-derived processes, as well as primary metabolic intermediates. The detection of these compounds at elevated temperatures suggests that controlled heating enabled access to metabolites that may not be readily observed under ambient conditions.

The biosynthetic pathways are summarized in Fig. 3B. By organizing VOCs according to their biosynthetic origin, this analysis not only clarifies the chemical diversity accessible via TP-SESI but also highlights the biological relevance of each class.

**Fig. 4:**
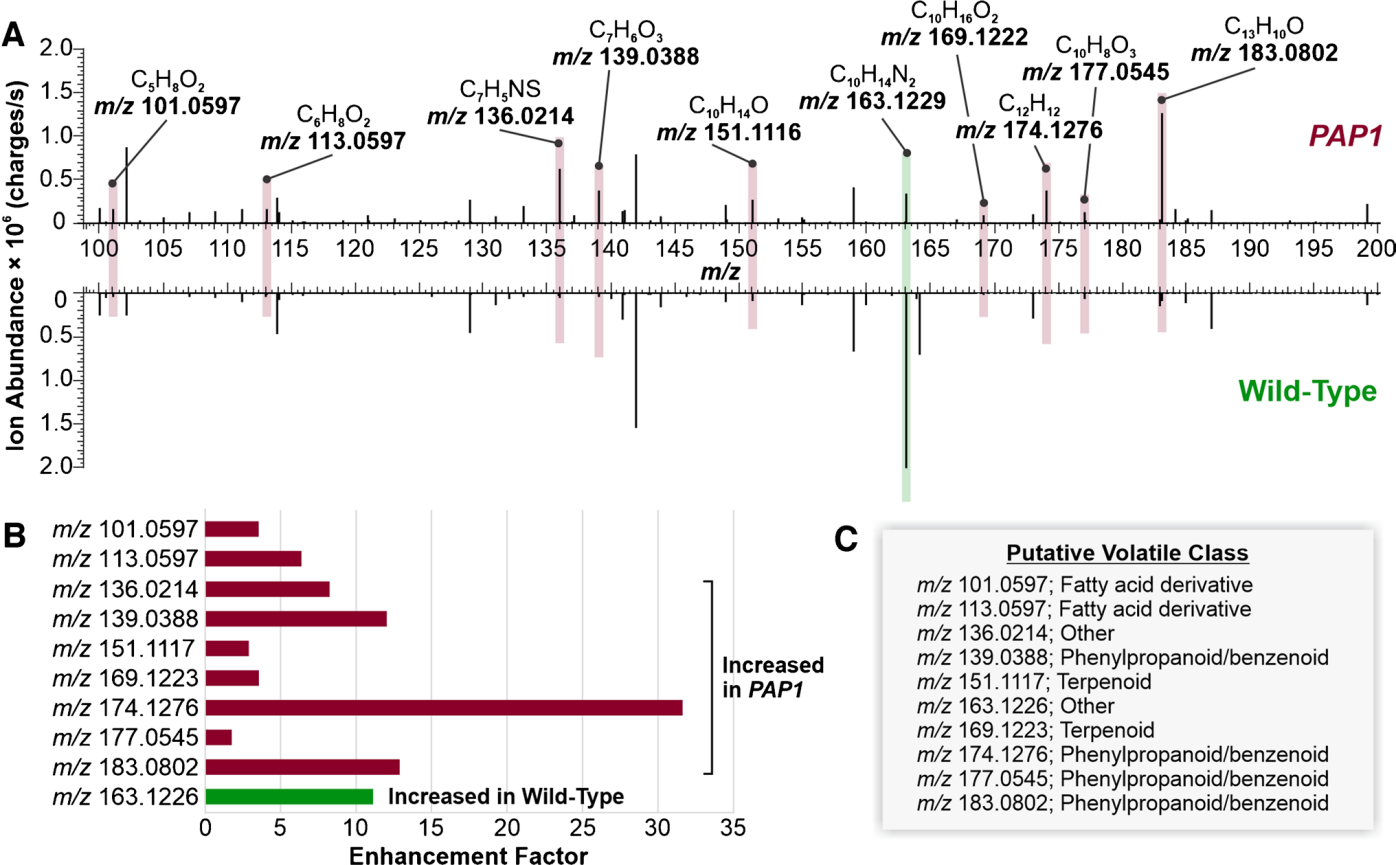
**A** Reflection mass spectra of 10 representative VOCs comparing *PAP1*-overexpressing plants (top) and wild-type plants (bottom). All VOCs are upregulated in *PAP1* except nicotine (*m*/*z* 163.1226), which is more abundant in wild-type plants. **B** Enhancement factor plot shows fold changes in VOC abundance between genotypes. This is calculated by the ratio of mean ion abundance of *PAP1* samples to wild-type. Red bars indicate upregulation in *PAP1*-overexpressing plants, while the green bar indicates higher abundance in wild-type plants. **C** Table of putative VOC annotations corresponding to detected *m/z* values and associated compound classes

We compared the mass spectra of *PAP1*-overexpressing and wild-type leaves at 55 °C to assess genotype-dependent differences in VOC emission within *m*/*z* 100–200 range. Figure 4A presents a reflection plot of 10 abundant VOCs in this range, selected to visualize the dominant features of the spectra. Of these, 9 analytes were consistently upregulated in *PAP1* leaves, while nicotine was downregulated, consistent with previous reports demonstrating that *PAP1 *overexpression can indirectly suppress certain alkaloid pathways [42]. The magnitude of these signals in *PAP1* is quantified in Fig. 4B, which displays enhancement factor comparisons for each VOC, following the same pattern. Enhancement factors were calculated as the ratio of mean ion abundance in *PAP1* samples to the mean ion abundance in wild-type samples. These show the majority of the *PAP1*-upregulated compounds exhibit increases of approximately half to a full order of magnitude relative to wild-type. Nicotine, in contrast, is over an order of magnitude downregulated, reinforcing mass spectra observations. The putative biosynthetic classification and for these 10 compounds is summarized in Fig. 4C. Most of the upregulated compounds in *PAP1* belong to the phenylpropanoid/benzenoid class, but all other classes are represented. These assignments are consistent with the known metabolic reprogramming driven by *PAP1* overexpression, including upregulation of flavonoids and anthocyanin precursors, as well as associated glycosylated and nitrogen-containing conjugates.

**Fig. 5:**
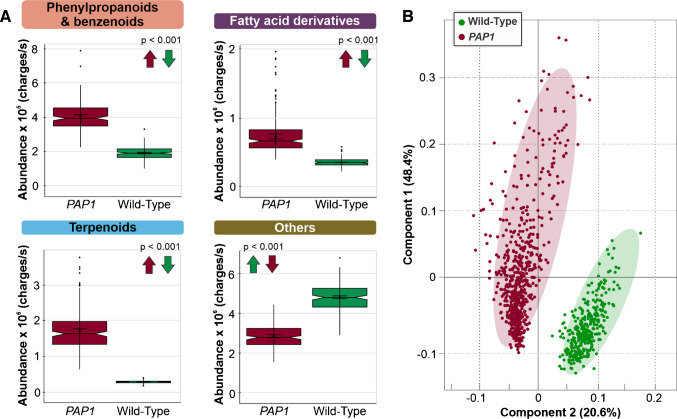
**A** Notched boxplots showing summed abundances of VOCs grouped by biosynthetic class. Terpenoids, fatty acids derivatives, and phenylpropanoids/benzenoids are significantly upregulated in *PAP1* leaves (red), while the “other” category (primarily nicotine), is enriched in wild-type leaves (green). The arrows show this pattern. All *p*-values were less than 0.001 showing strong statistical differences. **B** PCA of VOC profiles from wild-type and *PAP1*-overexpressing plants. The two genotypes cluster separately, with slightly greater dispersion observed within the *PAP1* group compared to wild-type, while still maintaining clear genotype-level separation

We performed statistical analysis demonstrating the effectiveness of TP-SESI in resolving genotype-dependent differences between *PAP1*-overexpressing and wild-type plants. Figure 5A presents notched boxplots of the summed abundances of each volatile class, revealing consistent upregulation of terpenoids, fatty acid derivatives, and phenylpropanoids/benzenoids in *PAP1 *leaves compared to wild-type. In contrast, the “other” category was more abundant in wild-type leaves, primarily driven by the presence of nicotine, consistent with previous reports [42]. All comparisons were highly statistically significant (*p* < 0.001), demonstrating that *PAP1* overexpression broadly enhances production of multiple volatile classes while modestly reducing select alkaloid-related compounds.

The genotype-dependent differences are further supported by principal component analysis (PCA) in Fig. 5B. PCA shows clear separation between *PAP1* and wild-type samples, with consistent grouping of measurements within each genotype. This pattern indicates that genotype is a dominant source of variance in the VOC profiles, while the compact clustering highlights the reproducibility and robustness of the TP-SESI workflow. To contextualize these genotype-dependent differences, the biosynthetic class assignments highlight that the separation observed in PCA is largely associated with the classes most affected by *PAP1* overexpression. Terpenoids, fatty acid derivatives, and phenylpropanoids/benzenoids all show higher abundance in *PAP1* leaves, consistent with the known role of *PAP1* in upregulating secondary metabolism, particularly the phenylpropanoid pathway. The “other” category, dominated by nicotine, is higher in wild-type leaves, reflecting that this alkaloid does not fall under the pathways directly influenced by *PAP1*.

## Conclusions

TP-SESI enabled high-resolution profiling of volatile and semi-volatile metabolites in *PAP1*-overexpressing and wild-type tobacco leaves. Controlled thermal desorption produced reproducible, statistically significant VOC signals, allowing detection of both dominant and less-volatile compounds. *PAP1*-overexpressing leaves showed clear upregulation of terpenoids, fatty acid derivatives, and phenylpropanoids/benzenoids, while nicotine was enriched in wild-type leaves. These results demonstrate that TP-SESI can resolve biologically meaningful, genotype-dependent metabolic differences and provides a robust platform for studying plant secondary metabolism.

## Supplementary Information

Below is the link to the electronic supplementary material.

ESM 1 Supplementary Material 1 (DOCM 551 KB)

## Data Availability

Data that supports the findings of this study are available from the authors upon reasonable request. Supplementary materials associated with this article are available with the published study.
