# Supplementary material for: Differentiation of wild-type and PAP1-overexpressing tobacco by volatile organic compound profiling using TP-SESI mass spectrometry
Source: Anal Bioanal Chem. 2026 Jun 20;418(17):5577–85. doi: 10.1007/s00216-026-06630-y (PMC13473073; doi:10.1007/s00216-026-06630-y)
Supplement: Supplementary file 1 [file 216_2026_6630_MOESM1_ESM.docm]

**Supporting Information**

**Differentiation of Wild-Type and PAP1-Overexpressing Tobacco by Volatile Organic Compound Profiling Using TP-SESI Mass Spectrometry**

Sarah M. Ashbacher^1^, De-Yu Xie^2^, and David C. Muddiman*^1,2^

*^1^Biological Imaging Laboratory for Disease and Exposure Research (BILDER), Department of Chemistry, North Carolina State University, Raleigh, NC 27695*

*^2^Department of Plant and Microbial Biology, North Carolina State University, Raleigh, NC 27695*

**Submitted to:** *Analytical and Bioanalytical Chemistry*

***Author for Correspondence**

David C. Muddiman, Ph.D.

Biological Imaging Laboratory for Disease and Exposure Research

Department of Chemistry

North Carolina State University

Email: [dcmuddim@ncsu.edu](mailto:dcmuddim@ncsu.edu)

**Table of Contents**

**Table S1.** Putative compound identification	S2

**Figure S1.** Spectral accuracy of the 10 representative VOCs	S8

**Figure S2.** EICs of blank controls	S9

**Figure S3.** Mass spectra of 3 anthocyanins in *PAP1*	S10

| **Molecular formula** | **Adduct** | ***m/z*** | **Mass error** | **MSM** | **Putative ID** | **Chemical category** | **Putative volatile class ID** |
| --- | --- | --- | --- | --- | --- | --- | --- |
| C_13_H_10_O | M+H | 183.0804 | -1.45 | 0.99 | benzophenone | benzophenone | phenylpropanoid/benzenoid |
| C_10_H_14_N_2_ | M+H | 163.1226 | -1.26 | 0.98 | nicotine | alkaloid | other |
|  |  |  |  |  | anabasine |  |  |
| C_12_H_12_ | M+NH_4_ | 174.1277 | -0.78 | 0.98 | dimethylnaphthalene | aromatic hydrocarbon | phenylpropanoid/benzenoid |
| C_8_H_12_O_2_ | M+H | 141.091 | -0.82 | 0.97 | 3-(3-Buten-1-yl)dihydro-2(3H)-furanone | lactone | fatty acid derivative |
| C_7_H_5_NS | M+H | 136.0215 | -0.74 | 0.97 | benzothiazole | heteroaromatic | other |
| C_5_H_8_O_2_ | M+H | 101.0597 | -0.58 | 0.95 | tiglic acid | short-chain fatty acid | fatty acid derivative |
|  |  |  |  |  | dihydro-3-methyl-2(3H)-furanone | cyclic ester |  |
|  |  |  |  |  | γ-valerolactone | lactone |  |
| C_12_H_18_O_4_ | M+H | 227.1278 | 0.05 | 0.94 | 12-Hydroxyjasmonic Acid | oxylipin | fatty acid derivative |
|  |  |  |  |  | tuberonic acid |  |  |
| C_10_H_14_O | M+H | 151.1117 | -0.7 | 0.92 | carvone | oxygenated monoterpene | terpenoid |
| C_6_H_8_O_2_ | M+H | 113.0597 | -0.57 | 0.92 | 5-Dimethyl-2(5H)-furanone | lactone (furanone) | fatty acid derivative |
| C_10_H_12_O | M+H | 149.0961 | -0.74 | 0.92 | cuminal | aromatic ketone | phenylpropanoid/benzenoid |
| C_20_H_30_ | M+H | 271.242 | 0.17 | 0.91 | abietatreine | diterpene | terpenoid |
| C_7_H_8_O_3_ | M+H | 141.0546 | -0.55 | 0.9 | methyltriacetic lactone | lactone; cyclic ester | fatty acid derivative |
| C_20_H_32_O | M+H | 289.2526 | 0.17 | 0.87 | ent-kaurenol | diterpenoid | terpenoid |
|  |  |  |  |  | isoraimonol |  |  |
| C_8_H_9_NO | M+H | 136.0757 | -0.79 | 0.86 | phenylacetaldoxime | aromatic oxime | phenylpropanoid/benzenoid |
| C_11_H_18_O_4_ | M+H | 215.1278 | 0 | 0.86 | 1-Methyl-3-(1-methylethyl)-1,2-cyclopentanedicarboxylic acid | cyclopentane | terpenoid |
| C_8_H_10_ | M+H | 107.0855 | -0.63 | 0.86 | ethylbenzene | aromatic hydrocarbon | phenylpropanoid/benzenoid |
|  |  |  |  |  | xylene |  |  |
| C_15_H_22_O_2_ | M+H | 235.1693 | -0.03 | 0.86 | 3-hydroxysolavetivone | sesquiterpene | terpenoid |
|  |  |  |  |  | tetradymol |  |  |
|  |  |  |  |  | vellerol |  |  |
| C_6_H_12_O_2_ | M+H | 117.091 | -0.9 | 0.85 | hexanoic acid | hexanoic acid | fatty acid derivative |
| C_6_H_6_O_3_ | M+H | 127.039 | -0.76 | 0.85 | 5-Hydroxymethyl-2-furaldehyde | sugar derivative | other |
|  |  |  |  |  | maltol |  |  |
| C_15_H_20_ | M+H | 201.1638 | -0.3 | 0.85 | α-/β-/γ-calacorene | sesquiterpene | terpenoid |
| C_7_H_6_O_3_ | M+H | 139.039 | -0.68 | 0.84 | 5-Acetylfurfural | furan aldehyde | phenylpropanoid/benzenoid |
| C_20_H_32_ | M+H | 273.2577 | 0.1 | 0.84 | cembrene | diterpene | terpenoid |
| C_10_H_16_O_2_ | M+H | 169.1223 | -0.66 | 0.83 | diosphenol | monoterpene | terpenoid |
|  |  |  |  |  | geranic acid |  |  |
| C_14_H_20_O_2_ | M+H | 221.1536 | -0.11 | 0.83 | 2-Phenethyl hexanoate | hexanoate derivative | fatty acid derivative |
| C_10_H_20_O | M+NH_4_ | 174.1852 | -1.23 | 0.83 | 3,7-Dimethyloct-2-en-1-ol | monoterpene | terpenoid |
|  |  |  |  |  | menthol |  |  |
| C_10_H_16_ | M+H | 137.1325 | -0.88 | 0.82 | limonene | monoterpene hydrocarbon | terpenoid |
|  |  |  |  |  | pinene |  |  |
| C_15_H_22_O | M+H | 219.1743 | -0.23 | 0.81 | solavetivone | sesquiterpene ketones | terpenoid |
|  |  |  |  |  | salanascone |  |  |
| C_13_H_22_O | M+H | 195.1743 | -0.15 | 0.81 | solanone | apocarotenoid | terpenoid |
| C_10_H_18_O | M+H | 155.143 | -1.09 | 0.8 | linalool | monoterpene alcohol | terpenoid |
|  |  |  |  |  | geraniol |  |  |
|  |  |  |  |  | nerol |  |  |
| C_14_H_22_O_2_ | M+H | 223.1693 | -0.15 | 0.8 | rishitin | sesquiterpene | terpenoid |
|  |  |  |  |  | 4,5-Epoxy-13-hydroxy-β-caryophyllene |  |  |
| C_18_H_32_O_4_ | M+H | 313.2373 | 0.12 | 0.79 | octadeca-9-Ene-1,18-Dioic-Acid | dicarboxylic acid | fatty acid derivative |
| C_12_H_22_O_2_ | M+H | 199.1693 | 0.12 | 0.78 | 3,7-dimethyloct-1-en-3-yl acetate | monoterpene ester | terpenoid |
| C_10_H_14_O_2_ | M+H | 167.1067 | -1.1 | 0.76 | thymohydroquinone | hydroquinone derivative | phenylpropanoid/benzenoid |
| C_9_H_13_N | M+H | 136.1121 | -1.51 | 0.76 | N-Ethyl-m-toluidine | aromatic amine | other |
| C_10_H_16_O | M+H | 153.1274 | -0.95 | 0.75 | β-cyclocitral | apocarotenoid | terpenoid |
| C_14_H_24_O | M+H | 209.19 | -0.14 | 0.75 | α-Methyl ionol | apocarotenoid | terpenoid |
|  |  |  |  |  | 4-(2,6,6-Trimethyl-2-cyclohexen-1-yl)-2-pentanone |  |  |
|  |  |  |  |  | 1,13-Tetradecadien-3-one |  |  |
| C_7_H_10_O | M+NH_4_ | 128.107 | -0.94 | 0.75 | 2,4-Heptadienal | unsaturated aldehyde | fatty acid derivative |
| C_9_H_14_O | M+H | 139.1117 | -0.75 | 0.75 | 2-Pentylfuran | linoleic acid derived | fatty acid derivative |
| C_13_H_24_O_2_ | M+H | 213.1849 | -0.15 | 0.73 | 3-Hydroxy-7,8-dihydro-β-ionol | apocarotenoid | terpenoid |
|  |  |  |  |  | tectoionol b |  |  |
| C_6_H_8_O_3_ | M+H | 129.0546 | -0.85 | 0.73 | osmundalactone | lactone | fatty acid derivative |
|  |  |  |  |  | sotolone |  |  |
| C_10_H_8_O_3_ | M+H | 177.0546 | -0.48 | 0.72 | 4-Methyl-7-hydroxycoumarin | coumarin | phenylpropanoid/benzenoid |
|  |  |  |  |  | Ayapanin |  |  |
|  |  |  |  |  | 6-Hydroxy-7-methyl-2H-chromen-2-one |  |  |
|  |  |  |  |  | (E)-3,4-Methylenedioxycinnamaldehyde |  |  |
| C_5_H_9_NO_2_ | M+H | 116.0706 | -0.89 | 0.72 | proline | amino acid | other |
| C_5_H_6_O_2_ | M+NH_4_ | 116.0706 | -0.89 | 0.72 | furfuryl alcohol | furan | fatty acid derivative |
|  |  |  |  |  | 3-Methyl-2(5H)-furanone | lactone |  |
| C_15_H_24_O_2_ | M+H | 237.1849 | -0.09 | 0.71 | capsidiol | sesquiterpene | terpenoid |
|  |  |  |  |  | lubimin |  |  |
| C_6_H_10_O_2_ | M+H | 115.0754 | -0.78 | 0.68 | 5-caprolactone | lactone | fatty acid derivative |
|  |  |  |  |  | tetrahydro-4-methyl-2H-pyran-2-one |  |  |
| C_10_H_15_NO | M+NH_4_ | 183.1492 | -1.59 | 0.68 | 1-(3-Methylbutyl)-2-formylpyrrole | nitrogen heterocycle | fatty acid derivative |
| C_9_H_19_NO | M+H | 158.1539 | -1.27 | 0.67 | isophorol | monoterpenoid ketone | terpenoid |
| C_9_H_16_O | M+NH_4_ | 158.1539 | -1.27 | 0.67 | (3E)-5-Ethyl-3-hepten-2-one | unsaturated ketone | fatty acid derivative |
| C_5_H_8_O_5_ | M+H | 149.0444 | 1.67 | 0.65 | citramalic acid | organic acid | other |
|  |  |  |  |  | 2-hydroxyglutaric acid |  |  |
| C_10_H_18_O | M+NH_4_ | 172.1696 | -1.16 | 0.64 | geraniol | monoterpene | terpenoid |
|  |  |  |  |  | nerol |  |  |
|  |  |  |  |  | linalool |  |  |
|  |  |  |  |  | citronellal |  |  |
| C_9_H_16_O_2_ | M+H | 157.1223 | -1.15 | 0.63 | γ-nonalactone | lactone | fatty acid derivative |
| C_9_H_11_NO | M+H | 150.0913 | -1.43 | 0.63 | 2-phenylethylaldoxime | nitrogen-containing alcohol | phenylpropanoid/benzenoid |
|  |  |  |  |  | 2-(3-Ethenylpyridin-4-yl)ethan-1-ol |  |  |
| C_9_H_8_O | M+NH_4_ | 150.0913 | -1.43 | 0.63 | cinnamaldehyde | classical phenylpropanoid | phenylpropanoid/benzenoid |
| C_13_H_20_O | M+H | 193.1587 | -0.34 | 0.59 | beta-ionone | apocarotenoid | terpenoid |
| C_14_H_22_O | M+H | 207.1743 | -0.1 | 0.58 | 2,4-Bis(1,1-dimethylethyl)phenol | alkylated phenol | phenylpropanoid/benzenoid |
| C_15_H_24_ | M+H | 205.1951 | -0.26 | 0.58 | caryophyllene | sesquiterpene | terpenoid |
| C_12_H_14_O_4_ | M+H | 223.0965 | -0.04 | 0.57 | 15-Nor-2-Acoren-4-One | sesquiterpene | terpenoid |
|  |  |  |  |  | 4-(6-methylhept-5-en-2-yl)cyclohex-2-en-1-one |  |  |
| C_8_H_15_NO | M+H | 142.1226 | -1.56 | 0.57 | hygrine | alkaloid | other |
| C_8_H_12_O | M+NH_4_ | 142.1226 | -1.56 | 0.57 | 1-(2-Methyl-2-cyclopenten-1-yl)ethanone | cyclopenenone | terpenoid |
|  |  |  |  |  | 3,4,4-Trimethyl-2-cyclopenten-1-one |  |  |
|  |  |  |  |  | 3,5,5-Trimethylcyclopent-2-en-1-one |  |  |
| C_5_H_8_O | M+NH_4_ | 102.0913 | -0.45 | 0.57 | 1-Penten-3-one | lipid oxidation ketones/GLV | fatty acid derivative |
|  |  |  |  |  | 3-Methyl-2-butenal |  |  |
|  |  |  |  |  | (E)-3-Penten-2-one |  |  |
| C_6_H_11_NO | M+H | 114.0913 | -0.68 | 0.56 | (2E,4E)-hexa-2,4-dienal | aldehyde/GLV | fatty acid derivative |
| C_6_H_8_O | M+NH_4_ | 114.0913 | -0.68 | 0.56 | Cyclohex-2-en-1-one | cyclohexenone | other |
| C_7_H_10_O_2_ | M+H | 127.0754 | -1.08 | 0.55 | 3-Ethyl-2-hydroxy-2-cyclopenten-1-one | cyclopentenone | fatty acid derivative |
| C_6_H_6_O_2_ | M+H | 111.0441 | -0.93 | 0.53 | 2-Acetylfuran | furan | fatty acid derivative |
|  |  |  |  |  | 5-Methylfurfural |  |  |
| C_9_H_14_O_2_ | M+H | 155.1067 | -0.8 | 0.53 | 4-Hydroxy-2,6,6-trimethyl-2-cyclohexen-1-one | apocarotenoid | terpenoid |
|  |  |  |  |  | 2-Acetyl-5-methylcyclohexan-1-one |  |  |
|  |  |  |  |  | 4-Hydroxy-3,5,5-trimethylcyclohex-2-enone |  |  |
| C_10_H_12_O_2_ | M+H | 165.091 | -1.39 | 0.53 | eugenol | classic phenylpropanoid | phenylpropanoid/benzenoid |
|  |  |  |  |  | isoeugenol |  |  |
|  |  |  |  |  | 2-phenylethyl acetate |  |  |
| C_10_H_14_ | M+H | 135.1168 | -1.23 | 0.52 | p-cymene | monoterpene hydrocarbon | terpenoid |
|  |  |  |  |  | p-mentha-1,5,8-triene |  |  |
| C_15_H_20_O | M+H | 217.1587 | -0.28 | 0.52 | dehydrosolanascone | Sesquiterpene ketones | terpenoid |
| C_9_H_12_O | M+H | 137.0961 | -1.18 | 0.52 | 1-(4-Methylphenyl)ethanol | benzenoid alcohol | phenylpropanoid/benzenoid |
|  |  |  |  |  | 2-Phenylpropan-1-ol |  |  |
|  |  |  |  |  | Phenylpropanol |  |  |
|  |  |  |  |  | 2-Ethylbenzyl alcohol |  |  |
| C_14_H_26_O_2_ | M+H | 227.2006 | -0.1 | 0.51 | myristoleic acid | ester/free fatty acid | fatty acid derivative |
|  |  |  |  |  | decyl 2-Methylprop-2-Enoate |  |  |
| C_7_H_6_O | M+NH_4_ | 124.0757 | -1.44 | 0.5 | benzaldehyde | benzenoid aldehyde | phenylpropanoid/benzenoid |
| C_7_H_9_NO | M+H | 124.0757 | -1.44 | 0.5 | 1-(1-Methyl-1H-pyrrol-3-yl)ethanone | pyrrol derivative | other |
| C_8_H_14_O_4_ | M+H | 175.0965 | -1.12 | 0.5 | suberic acid | dicarboxylic acid | fatty acid derivative |
|  |  |  |  |  | diethylsuccinate |  |  |
| C_7_H_7_NO | M+H | 122.06 | -1.21 | 0.49 | 2-/3-/4-Acetylpyridine | aromatic N-heterocycle | phenylpropanoid/benzenoid |
| C_8_H_8_ | M+H | 105.0699 | -1.09 | 0.48 | styrene | aromatic hydrocarbon (benzeonoid) | phenylpropanoid/benzenoid |
| C_10_H_18_O_2_ | M+H | 171.138 | -0.98 | 0.48 | linalool oxide | monoterpene | terpenoid |
|  |  |  |  |  | (2E,6S)-Dimethyl-2,7-octadiene-1,6-diol |  |  |
| C_8_H_12_O | M+H | 125.0961 | -0.96 | 0.47 | 3,4,4-Trimethyl-2-cyclopenten-1-one | apocarotenoid | terpenoid |
|  |  |  |  |  | 4,5-Dimethyl-2-cyclohexen-1-one |  |  |
|  |  |  |  |  | 1-(2-Methyl-2-cyclopenten-1-yl)ethanone |  |  |
|  |  |  |  |  | 3,5,5-Trimethylcyclopent-2-en-1-one |  |  |
|  |  |  |  |  | 3,5-Dimethylcyclohex-2-en-1-one |  |  |
| C_8_H_14_O_2_ | M+H | 143.1067 | -1.16 | 0.47 | (3E)-hexenyl acetate | green leaf volatile | fatty acid derivative |
| C_10_H_10_O_2_ | M+H | 163.0754 | -0.5 | 0.47 | 5-Hydroxy-3-methyl-1-indanone | phenylpropanoid/benzenoid | phenylpropanoid/benzenoid |
|  |  |  |  |  | Prop-2-en-1-yl benzoate |  |  |
|  |  |  |  |  | (E)-3-(2-Methoxyphenyl)Prop-2-enal |  |  |
|  |  |  |  |  | 4-Methoxycinnamaldehyde |  |  |
| C_22_H_42_O_4_ | M+H | 371.3156 | 0.31 | 0.47 | 1,22-Docosane dioic acid | dicarboxylic acid | fatty acid derivative |
| C_13_H_22_O_3_ | M+H | 227.1642 | -0.1 | 0.46 | blumenol B | apocarotenoid | terpenoid |
|  |  |  |  |  | methyl dihydrojasmonate |  |  |

**Table S1**. Putative compound annotations used for data analysis. The table includes molecular formula, detected adduct, observed *m/z* value, mass measurement accuracy (MMA), METASPACE metabolite-signal match (MSM) score, putative identification(s), chemical category, and assigned volatile class. The MSM score is a confidence metric generated in METASPACE that helps identify and rank potential metabolite assignments. It ranges from 0 to 1, which higher values indicating a stronger match between the measured signal and the annotated metabolite. Annotations with an MSM score below 0.45 were excluded. All anntations retained for analysis met an FDR threshold of $\leq$ 10%. This value represents the estimated proportion of false-positive annotations among accepted matches. The initial list of annotations was generated using METASPACE and subsequently cross-validated against the vocBinBase and KNApSAcK databases. To be included in downstream analysis, each annotation was required to appear in at least two of the three databases. For molecular formulas associated with multiple possible compound identifications, all plausible candidates are reported along with their corresponding chemical category and volatile class. Annotations are presented in descending order of MSM score from highest to lowest confidence level.

**Figure S1.** Spectral accuracy plots for isotopic distribution validation of representative VOC annotations. The plots show the raw isotopic peak abundances for the monoisotopic (M), M+1, and M+2 peaks of 10 VOCs detected in PAP1 plant. The spectra shown corresponds to a single representative scan selected to illustrate isotopic agreement. For each compound, the solid line represents the experimentally observed isotopic abundances derived from the measured mass spectra, while the red dots indicate the corresponding theoretical isotopic abundances calculated based on elemental composition. Close agreement between observed and theoretical values supports accurate molecular formula assignment and strengthens confidence in the putative metabolite identifications.

**Figure S2.** Extracted ion chromatograms (EICs) for the 10 representative analytes acquired from procedural blank controls. Blank measurements consisted of **(A)** ESI acquired immediately after removal of the plant sample from the heated stage and **(B)** double-sided tape mounted on a blank glass slide under the same temperature program used for TP-SESI experiments. EICs represent the average of three replicate measurements. No persistent VOC features corresponding to the plant-derived analytes were detected in the controls. A low-abundance sporadic signal at m/z 139.0388 was occasionally observed in blank ESI measurements.

**Figure S3.** Representative IR-MALDESI mass spectra showing anthocyanins detected in PAP1-overexpressing tobacco leaves. Spectra represent the average of three replicate measurements and include **(A)** cyanidin, **(B)** delphinidin, and **(C)** malvidin monoisotopic (M) and M+1 peaks. These anthocyanin signals were detected in PAP1 samples but were absent in wild-type controls, consistent with the visible pigmentation phenotype shown in Figure 1 in the main text.
